# Polymorphic form II of 4,4′-methyl­enebis(benzene­sulfonamide)

**DOI:** 10.1107/S1600536810021409

**Published:** 2010-06-09

**Authors:** Thomas Gelbrich, Mairi F. Haddow, Ulrich J. Griesser

**Affiliations:** a Institute of Pharmacy, University of Innsbruck, Innrain 52, 6020 Innsbruck, Austria

## Abstract

In the title compound, C_13_H_14_N_2_O_4_S_2_ (alternative names: diphenyl­methane-4,4′-disulfonamide, nirexon, CRN: 535–66-0), the two benzene rings form a dihedral angle of 70.8 (1)°. There are two sets of shorter (H⋯O < 2.1 Å) and longer (H⋯O > 2.4 Å) N—H⋯O hydrogen bonds per sulfonamide NH_2_ group, which together result in hydrogen-bonded sheets parallel (102). Adjacent sheets are connected to one another by an additional N—H⋯N inter­action so that a three-dimensional network of hydrogen-bonded mol­ecules is formed. The investigated polymorph is identical with the form II previously described by Kuhnert-Brandstätter & Moser [(1981). *Mikrochim. Acta*, **75**, 421–440].

## Related literature

For the polymorphism of diphenyl­methane-4,4′-disulfonamide, see Kuhnert-Brandstätter & Moser (1981 ▶); Kuhnert-Brandstätter & Wunsch (1969 ▶).
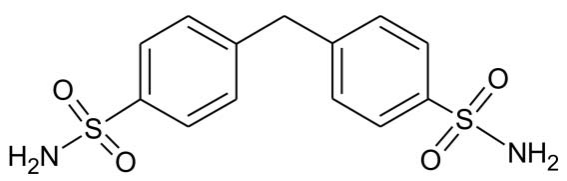


## Experimental

### 

#### Crystal data

C_13_H_14_N_2_O_4_S_2_
*M* *_r_* = 326.38
Monoclinic, 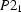

*a* = 10.8251 (5) Å
*b* = 5.0791 (3) Å
*c* = 12.6912 (5) Å
β = 90.931 (3)°
*V* = 697.69 (6) Å^3^
*Z* = 2
Mo *K*α radiation
μ = 0.40 mm^−1^
*T* = 120 K
0.25 × 0.1 × 0.05 mm

#### Data collection

Bruker–Nonius Roper CCD camera on κ-goniostat diffractometer
Absorption correction: multi-scan (*SADABS*; Sheldrick, 2007 ▶) *T*_min_ = 0.907, *T*_max_ = 0.980
7877 measured reflections
2438 independent reflections
2152 reflections with *I* > 2σ(*I*)
*R*_int_ = 0.054

#### Refinement

*R*[*F* ^2^ > 2σ(*F* ^2^)] = 0.038
*wR*(*F* ^2^) = 0.087
*S* = 1.06
2438 reflections
216 parameters
5 restraints
H atoms treated by a mixture of independent and constrained refinement
Δρ_max_ = 0.26 e Å^−3^
Δρ_min_ = −0.46 e Å^−3^
Absolute structure: Flack (1983 ▶), 904 Friedel pairs
Flack parameter: −0.18 (9)

### 

Data collection: *COLLECT* (Hooft, 1998 ▶); cell refinement: *SCALEPACK* (Otwinowski & Minor, 1997 ▶); data reduction: *DENZO* (Otwinowski & Minor, 1997 ▶) and *SCALEPACK*; program(s) used to solve structure: *SHELXS97* (Sheldrick, 2008 ▶); program(s) used to refine structure: *SHELXL97* (Sheldrick, 2008 ▶); molecular graphics: *XP* in *SHELXTL* (Sheldrick, 2008 ▶) and *Mercury* (Macrae *et al.*, 2006 ▶); software used to prepare material for publication: *publCIF* (Westrip, 2010 ▶).

## Supplementary Material

Crystal structure: contains datablocks global, I. DOI: 10.1107/S1600536810021409/im2209sup1.cif

Structure factors: contains datablocks I. DOI: 10.1107/S1600536810021409/im2209Isup2.hkl

Additional supplementary materials:  crystallographic information; 3D view; checkCIF report

## Figures and Tables

**Table 1:** Hydrogen-bond geometry (Å, °)

*D*—H⋯*A*	*D*—H	H⋯*A*	*D*⋯*A*	*D*—H⋯*A*
N1—H1⋯O1^i^	0.88 (2)	2.09 (2)	2.960 (4)	168 (4)
N1—H2⋯O1^ii^	0.89 (2)	2.42 (3)	3.166 (4)	142 (3)
N1—H2⋯O4^iii^	0.89 (2)	2.53 (3)	3.116 (4)	124 (3)
N2—H3⋯O3^iv^	0.88 (2)	2.06 (2)	2.898 (3)	159 (3)
N2—H4⋯N1^v^	0.88 (2)	2.50 (3)	3.182 (3)	135 (3)
N2—H4⋯O4^vi^	0.88 (2)	2.50 (3)	3.149 (4)	131 (3)

Symmetry codes: (i) 
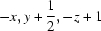
; (ii) 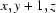; (iii) 
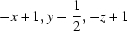
; (iv) 
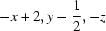
; (v) 
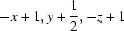
; (vi) 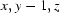.

**Fig. 1.**
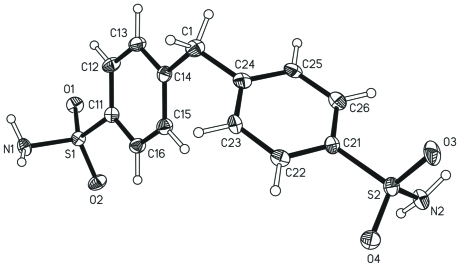
The molecular structure with displacement ellipsoids drawn at the 50% probability level and hydrogen atoms shown as spheres of arbitrary size.

**Fig. 2.**
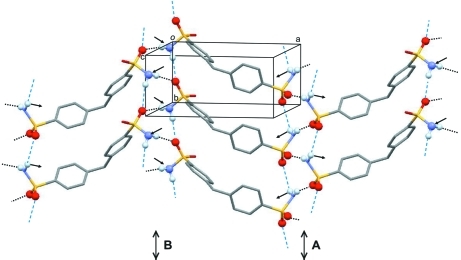
Portion of a single hydrogen bonded sheet parallel to (102) and generated by shorter N—H···O bonds (dotted lines) and longer N—H···O bonds (dashed lines). N—H···N bonds (arrows) connect to neighbouring sheets. O, H and N atoms directly involved in hydrogen bonds are drawn as balls.

## supplementary crystallographic information

### Comment

The polymorphic behaviour of the title compound, a carbonic anhydrase inhibitor with diuretic effects, was described by Kuhnert-Brandstätter & Moser (1981) and Kuhnert-Brandstätter & Wunsch (1969), who identified five distinct polymorphic modifications. The commercial product contained form II (mp. 172–174 °C), which was also found to be the stable polymorph at room temperature. The identity of the crystals investigated in the present study with form II reported by Kuhnert-Brandstätter & Moser (1981) was established by thermomicroscopy and IR spectroscopy. The asymmetric unit contains a single molecule (Fig. 1). Its central plane, defined by S1, S2, C1, C11, C14, C21 and C24, forms angles of 21.8 (1)° and 78.7 (1)° with the mean planes of the two phenyl rings C11 > C16 and C21 > C26, respectively. The two sulfonamide groups are differently oriented with respect to the adjacent phenyl ring, so that the corresponding torsion angles N1—S1—C1—C24 and N2—S2—C1—C14 are 115.6 (2)° and 53.3 (2)°, respectively. Each molecule is connected to four other molecules by four short N—H···O bonds (H···O < 2.1 Å, see Table 2) so that an H-bonded sheet parallel to (102) is formed. The symmetry operation between molecules linked by these primary interactions involving one H-bond donor and one acceptor site of each sulfonamide group is a twofold screw axis. There are two additional N—H···O contacts (H···O > 2.4 Å, see Table 2) within the same plane so that all four NH hydrogen bond donor sites are employed once. These secondary N—H···O bonds link molecules related by a translation along [010]. The O3 and O4 sites of one sulfonyl group accept one H-bond each, while in the group O1 accepts two H-bonds. Thus, the sulfonamide groups of molecules in the hydrogen bonded sheet form two distinct chains of fused rings both propagating parallel to [010]. One chain consists of ten-membered rings with two H-bond donor and two acceptor sites (labeled 'A' in Fig. 2), and the other is generated from 9-membered rings with two three H-bond donor and two acceptor sites ('B'). Each of the N—H···O sheets is linked to two neighbouring sheets by a N2—H···N1(-*x* + 1, *y* + 1/2, -*z* + 1) interaction (represented by arrows in Fig. 2). Thus, this crystal structure contains a three-dimensional network of N—H···O and N—H···N bonded molecules of diphenylmethane-4,4'-disulfonamide.

### Experimental

The crystals for this study were obtained from a commercial sample of diphenylmethane-4,4'-disulfonamide (Farbenfabriken Bayer AG, Leverkusen).

### Refinement

All H atoms were identified in a difference map. H atoms bonded to secondary CH_2_ (C—H = 0.99 Å) and aromatic carbon atoms (C—H = 0.95 Å) were positioned geometrically_. _Hydrogen atoms attached to N were refined with restrained distances [N—H = 0.88 (12) Å].The *U*_iso_ parameters of all H atoms were refined freely.

### Crystal data

**Table:**

C_13_H_14_N_2_O_4_S_2_	*F*(000) = 340
*M**_r_* = 326.38	*D*_x_ = 1.554 Mg m^−^^3^
Monoclinic, *P*2_1_	Mo *K*α radiation, λ = 0.71073 Å
Hall symbol: P 2yb	Cell parameters from 6304 reflections
*a* = 10.8251 (5) Å	θ = 2.9–26.0°
*b* = 5.0791 (3) Å	µ = 0.40 mm^−^^1^
*c* = 12.6912 (5) Å	*T* = 120 K
β = 90.931 (3)°	Needle, colourless
*V* = 697.69 (6) Å^3^	0.25 × 0.1 × 0.05 mm
*Z* = 2	

### Data collection

**Table:**

Bruker–Nonius Roper CCD camera on κ-goniostat diffractometer	2438 independent reflections
Radiation source: Bruker–Nonius FR591 rotating anode	2152 reflections with *I* > 2σ(*I*)
graphite	*R*_int_ = 0.054
Detector resolution: 9.091 pixels mm^-1^	θ_max_ = 26.0°, θ_min_ = 3.7°
φ & ω scans	*h* = −13→12
Absorption correction: multi-scan (*SADABS*; Sheldrick, 2007)	*k* = −5→6
*T*_min_ = 0.907, *T*_max_ = 0.980	*l* = −15→15
7877 measured reflections	

### Refinement

**Table:**

Refinement on *F*^2^	Secondary atom site location: difference Fourier map
Least-squares matrix: full	Hydrogen site location: inferred from neighbouring sites
*R*[*F*^2^ > 2σ(*F*^2^)] = 0.038	H atoms treated by a mixture of independent and constrained refinement
*wR*(*F*^2^) = 0.087	*w* = 1/[σ^2^(*F*_o_^2^) + (0.0361*P*)^2^ + 0.182*P*] where *P* = (*F*_o_^2^ + 2*F*_c_^2^)/3
*S* = 1.06	(Δ/σ)_max_ = 0.001
2438 reflections	Δρ_max_ = 0.26 e Å^−^^3^
216 parameters	Δρ_min_ = −0.46 e Å^−^^3^
5 restraints	Absolute structure: Flack (1983), 904 Friedel pairs
Primary atom site location: structure-invariant direct methods	Flack parameter: −0.18 (9)

### Special details

**Table:**

Geometry. All e.s.d.'s (except the e.s.d. in the dihedral angle between two l.s. planes) are estimated using the full covariance matrix. The cell e.s.d.'s are taken into account individually in the estimation of e.s.d.'s in distances, angles and torsion angles; correlations between e.s.d.'s in cell parameters are only used when they are defined by crystal symmetry. An approximate (isotropic) treatment of cell e.s.d.'s is used for estimating e.s.d.'s involving l.s. planes.
Refinement. Refinement of *F*^2^ against ALL reflections. The weighted *R*-factor *wR* and goodness of fit *S* are based on *F*^2^, conventional *R*-factors *R* are based on *F*, with *F* set to zero for negative *F*^2^. The threshold expression of *F*^2^ > σ(*F*^2^) is used only for calculating *R*-factors(gt) *etc*. and is not relevant to the choice of reflections for refinement. *R*-factors based on *F*^2^ are statistically about twice as large as those based on *F*, and *R*- factors based on ALL data will be even larger.

### Fractional atomic coordinates and isotropic or equivalent isotropic displacement parameters (Å^2^)

**Table:**

	*x*	*y*	*z*	*U*_iso_*/*U*_eq_	
S1	0.20189 (6)	−0.23188 (15)	0.54432 (5)	0.01593 (18)	
S2	0.88754 (6)	0.73072 (17)	0.14221 (5)	0.01695 (19)	
O1	0.1335 (2)	−0.4632 (4)	0.51332 (16)	0.0205 (5)	
O2	0.30683 (18)	−0.2579 (6)	0.61295 (14)	0.0238 (5)	
O3	0.90887 (19)	0.8314 (5)	0.03829 (15)	0.0249 (6)	
O4	0.90645 (19)	0.9060 (4)	0.23016 (16)	0.0208 (5)	
N1	0.1033 (3)	−0.0417 (6)	0.6023 (2)	0.0189 (6)	
H1	0.035 (3)	−0.039 (10)	0.563 (3)	0.053 (13)*	
H2	0.129 (3)	0.124 (4)	0.608 (3)	0.035 (11)*	
N2	0.9759 (2)	0.4794 (6)	0.1575 (2)	0.0197 (6)	
H3	0.994 (3)	0.410 (7)	0.0960 (18)	0.030 (10)*	
H4	0.965 (3)	0.381 (7)	0.213 (2)	0.042 (12)*	
C1	0.3517 (3)	0.3849 (7)	0.1577 (2)	0.0208 (7)	
H1A	0.3319	0.2784	0.0943	0.024 (8)*	
H1B	0.2990	0.5441	0.1550	0.028 (10)*	
C11	0.2504 (3)	−0.0654 (6)	0.4304 (2)	0.0169 (7)	
C12	0.1806 (3)	−0.0834 (7)	0.3372 (2)	0.0212 (7)	
H12	0.1097	−0.1934	0.3335	0.021 (8)*	
C13	0.2160 (3)	0.0610 (7)	0.2502 (2)	0.0200 (7)	
H13	0.1696	0.0470	0.1863	0.032 (10)*	
C14	0.3184 (2)	0.2261 (7)	0.2547 (2)	0.0164 (6)	
C15	0.3882 (2)	0.2386 (8)	0.3479 (2)	0.0189 (6)	
H15	0.4599	0.3463	0.3514	0.045 (12)*	
C16	0.3538 (3)	0.0945 (7)	0.4360 (2)	0.0188 (7)	
H16	0.4011	0.1058	0.4995	0.026 (9)*	
C21	0.7330 (3)	0.6228 (6)	0.1479 (2)	0.0151 (6)	
C22	0.6511 (2)	0.7639 (7)	0.2093 (2)	0.0174 (6)	
H22	0.6786	0.9108	0.2497	0.037 (11)*	
C23	0.5271 (3)	0.6854 (6)	0.2106 (2)	0.0179 (7)	
H23	0.4702	0.7814	0.2520	0.021 (8)*	
C24	0.4857 (3)	0.4701 (6)	0.1528 (2)	0.0170 (7)	
C25	0.5703 (3)	0.3302 (7)	0.0928 (2)	0.0183 (7)	
H25	0.5432	0.1810	0.0536	0.030 (10)*	
C26	0.6932 (3)	0.4053 (7)	0.0892 (2)	0.0187 (7)	
H26	0.7498	0.3100	0.0473	0.043 (11)*	

### Atomic displacement parameters (Å^2^)

**Table:**

	*U*^11^	*U*^22^	*U*^33^	*U*^12^	*U*^13^	*U*^23^
S1	0.0169 (3)	0.0134 (4)	0.0174 (3)	−0.0005 (4)	0.0005 (2)	0.0018 (4)
S2	0.0179 (4)	0.0157 (4)	0.0174 (3)	−0.0004 (4)	0.0033 (3)	0.0020 (4)
O1	0.0255 (12)	0.0093 (11)	0.0268 (12)	−0.0053 (10)	0.0004 (9)	−0.0009 (10)
O2	0.0203 (10)	0.0268 (13)	0.0241 (10)	−0.0016 (12)	−0.0057 (8)	0.0087 (13)
O3	0.0256 (12)	0.0306 (15)	0.0187 (10)	0.0016 (10)	0.0085 (8)	0.0113 (10)
O4	0.0222 (11)	0.0154 (12)	0.0249 (11)	−0.0026 (10)	0.0013 (9)	−0.0060 (10)
N1	0.0232 (15)	0.0143 (15)	0.0193 (14)	0.0003 (13)	0.0061 (11)	−0.0001 (12)
N2	0.0231 (14)	0.0191 (16)	0.0170 (14)	0.0058 (13)	0.0049 (11)	0.0032 (12)
C1	0.0209 (16)	0.0217 (18)	0.0196 (16)	−0.0019 (15)	−0.0019 (12)	0.0066 (14)
C11	0.0178 (15)	0.0145 (16)	0.0183 (14)	0.0002 (14)	0.0012 (12)	0.0014 (13)
C12	0.0173 (16)	0.0217 (19)	0.0246 (16)	−0.0055 (15)	−0.0045 (12)	0.0016 (15)
C13	0.0184 (16)	0.0229 (18)	0.0186 (15)	−0.0020 (15)	−0.0032 (12)	0.0005 (14)
C14	0.0155 (14)	0.0149 (16)	0.0189 (13)	0.0007 (15)	0.0011 (10)	0.0006 (15)
C15	0.0176 (14)	0.0204 (16)	0.0188 (13)	−0.0024 (17)	0.0009 (11)	0.0022 (17)
C16	0.0178 (16)	0.0191 (17)	0.0194 (15)	−0.0032 (14)	−0.0010 (12)	−0.0008 (13)
C21	0.0169 (15)	0.0134 (16)	0.0153 (13)	0.0005 (13)	0.0031 (11)	0.0024 (13)
C22	0.0228 (15)	0.0124 (15)	0.0169 (13)	−0.0014 (15)	−0.0009 (11)	0.0002 (16)
C23	0.0184 (15)	0.0181 (18)	0.0174 (14)	0.0015 (13)	0.0054 (12)	−0.0014 (13)
C24	0.0222 (16)	0.0168 (17)	0.0118 (14)	0.0009 (14)	−0.0020 (12)	0.0076 (13)
C25	0.0244 (16)	0.0183 (18)	0.0123 (13)	−0.0037 (13)	−0.0008 (12)	−0.0027 (12)
C26	0.0245 (16)	0.0153 (17)	0.0163 (14)	0.0028 (15)	0.0019 (12)	0.0002 (14)

### Geometric parameters (Å, °)

**Table:**

S1—O2	1.4263 (19)	C12—C13	1.385 (4)
S1—O1	1.440 (2)	C12—H12	0.9500
S1—N1	1.624 (3)	C13—C14	1.390 (4)
S1—C11	1.762 (3)	C13—H13	0.9500
S2—O3	1.437 (2)	C14—C15	1.395 (4)
S2—O4	1.440 (2)	C15—C16	1.391 (4)
S2—N2	1.605 (3)	C15—H15	0.9500
S2—C21	1.763 (3)	C16—H16	0.9500
N1—H1	0.881 (19)	C21—C22	1.389 (4)
N1—H2	0.887 (19)	C21—C26	1.397 (4)
N2—H3	0.883 (18)	C22—C23	1.401 (4)
N2—H4	0.875 (19)	C22—H22	0.9500
C1—C24	1.516 (4)	C23—C24	1.387 (4)
C1—C14	1.520 (4)	C23—H23	0.9500
C1—H1A	0.9900	C24—C25	1.395 (4)
C1—H1B	0.9900	C25—C26	1.386 (4)
C11—C16	1.384 (4)	C25—H25	0.9500
C11—C12	1.396 (4)	C26—H26	0.9500
			
O2—S1—O1	119.49 (15)	C12—C13—C14	121.1 (3)
O2—S1—N1	107.52 (14)	C12—C13—H13	119.4
O1—S1—N1	105.68 (14)	C14—C13—H13	119.4
O2—S1—C11	107.49 (13)	C13—C14—C15	118.9 (3)
O1—S1—C11	109.03 (14)	C13—C14—C1	119.1 (3)
N1—S1—C11	107.02 (15)	C15—C14—C1	122.0 (3)
O3—S2—O4	117.93 (14)	C16—C15—C14	120.6 (3)
O3—S2—N2	106.86 (13)	C16—C15—H15	119.7
O4—S2—N2	108.72 (13)	C14—C15—H15	119.7
O3—S2—C21	108.36 (13)	C11—C16—C15	119.7 (3)
O4—S2—C21	106.48 (13)	C11—C16—H16	120.2
N2—S2—C21	108.18 (15)	C15—C16—H16	120.2
S1—N1—H1	108 (3)	C22—C21—C26	120.9 (3)
S1—N1—H2	114 (2)	C22—C21—S2	118.5 (2)
H1—N1—H2	107 (4)	C26—C21—S2	120.6 (2)
S2—N2—H3	111 (2)	C21—C22—C23	118.7 (3)
S2—N2—H4	118 (3)	C21—C22—H22	120.6
H3—N2—H4	121 (4)	C23—C22—H22	120.6
C24—C1—C14	115.1 (2)	C24—C23—C22	121.3 (3)
C24—C1—H1A	108.5	C24—C23—H23	119.4
C14—C1—H1A	108.5	C22—C23—H23	119.4
C24—C1—H1B	108.5	C23—C24—C25	118.8 (3)
C14—C1—H1B	108.5	C23—C24—C1	120.2 (3)
H1A—C1—H1B	107.5	C25—C24—C1	120.9 (3)
C16—C11—C12	120.5 (3)	C26—C25—C24	121.1 (3)
C16—C11—S1	119.5 (2)	C26—C25—H25	119.4
C12—C11—S1	120.0 (2)	C24—C25—H25	119.4
C13—C12—C11	119.2 (3)	C25—C26—C21	119.2 (3)
C13—C12—H12	120.4	C25—C26—H26	120.4
C11—C12—H12	120.4	C21—C26—H26	120.4

### Hydrogen-bond geometry (Å, °)

**Table:**

*D*—H···*A*	*D*—H	H···*A*	*D*···*A*	*D*—H···*A*
N1—H1···O1^i^	0.88 (2)	2.09 (2)	2.960 (4)	168 (4)
N1—H2···O1^ii^	0.89 (2)	2.42 (3)	3.166 (4)	142 (3)
N1—H2···O4^iii^	0.89 (2)	2.53 (3)	3.116 (4)	124 (3)
N2—H3···O3^iv^	0.88 (2)	2.06 (2)	2.898 (3)	159 (3)
N2—H4···N1^v^	0.88 (2)	2.50 (3)	3.182 (3)	135 (3)
N2—H4···O4^vi^	0.88 (2)	2.50 (3)	3.149 (4)	131 (3)

Symmetry codes: (i) −*x*, *y*+1/2, −*z*+1; (ii) *x*, *y*+1, *z*; (iii) −*x*+1, *y*−1/2, −*z*+1; (iv) −*x*+2, *y*−1/2, −*z*; (v) −*x*+1, *y*+1/2, −*z*+1; (vi) *x*, *y*−1, *z*.
